# Supplementary figures and images for: The effects of sustained fitness improvement on the gut microbiome: A longitudinal, repeated measures case‐study approach
Source: Transl Sports Med. 2020 Dec 13;4(2):174–92. doi: 10.1002/tsm2.215 (PMC8317196; doi:10.1002/tsm2.215)

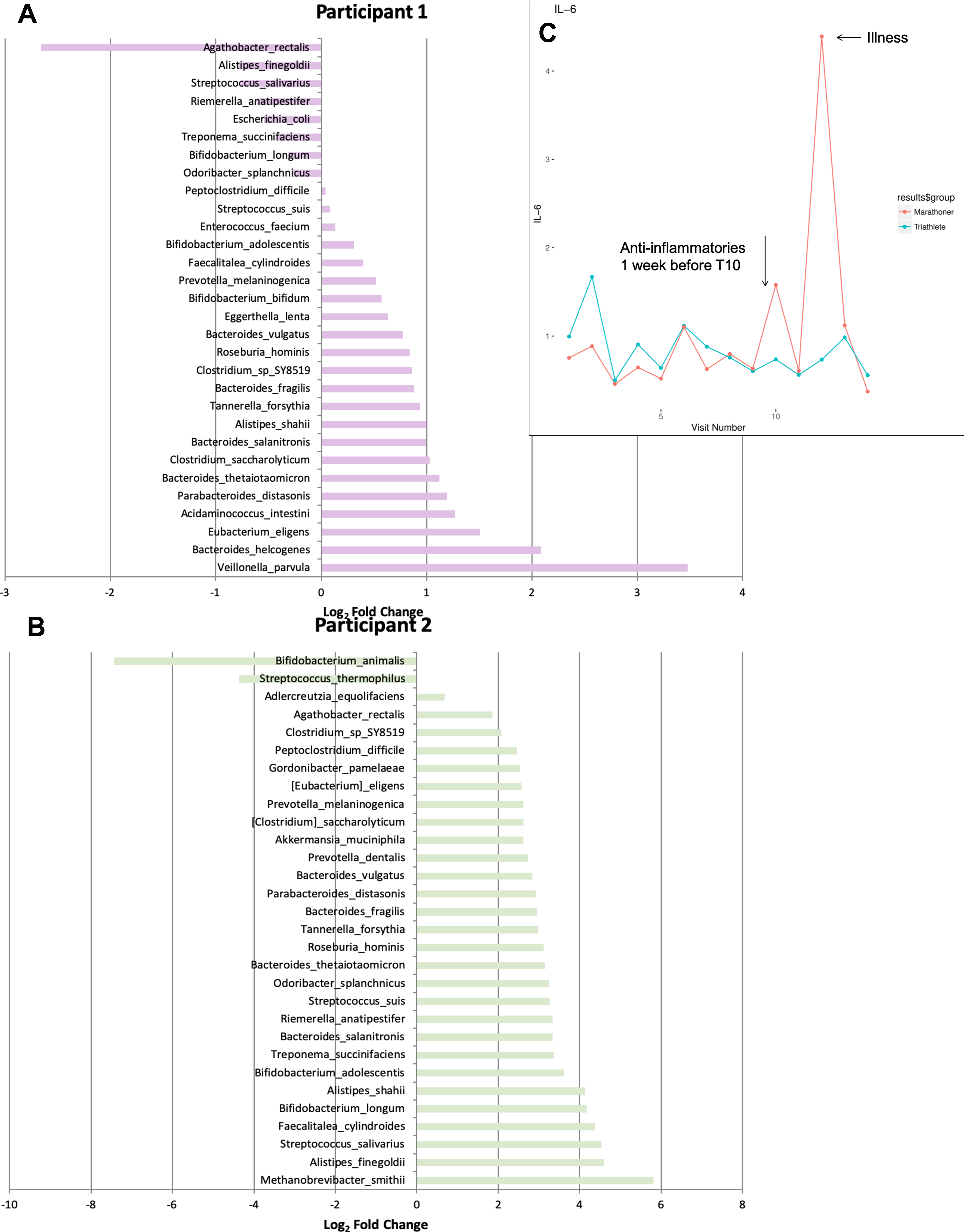

Supplement: Supplementary file 1 — Fig S1 [file TSM2-4-174-s002.tif]

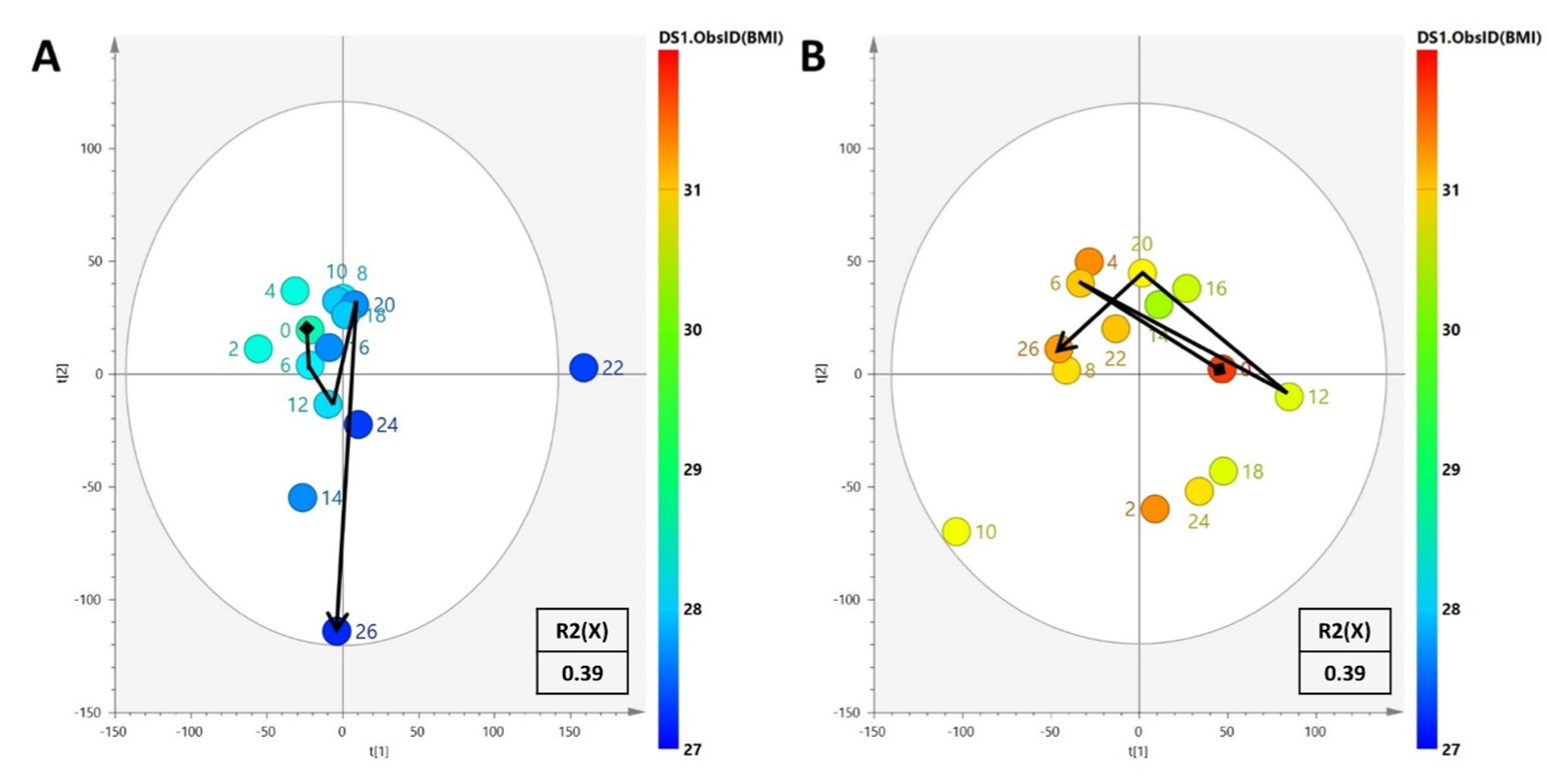

Supplement: Supplementary file 2 — Fig S2 [file TSM2-4-174-s004.tif]

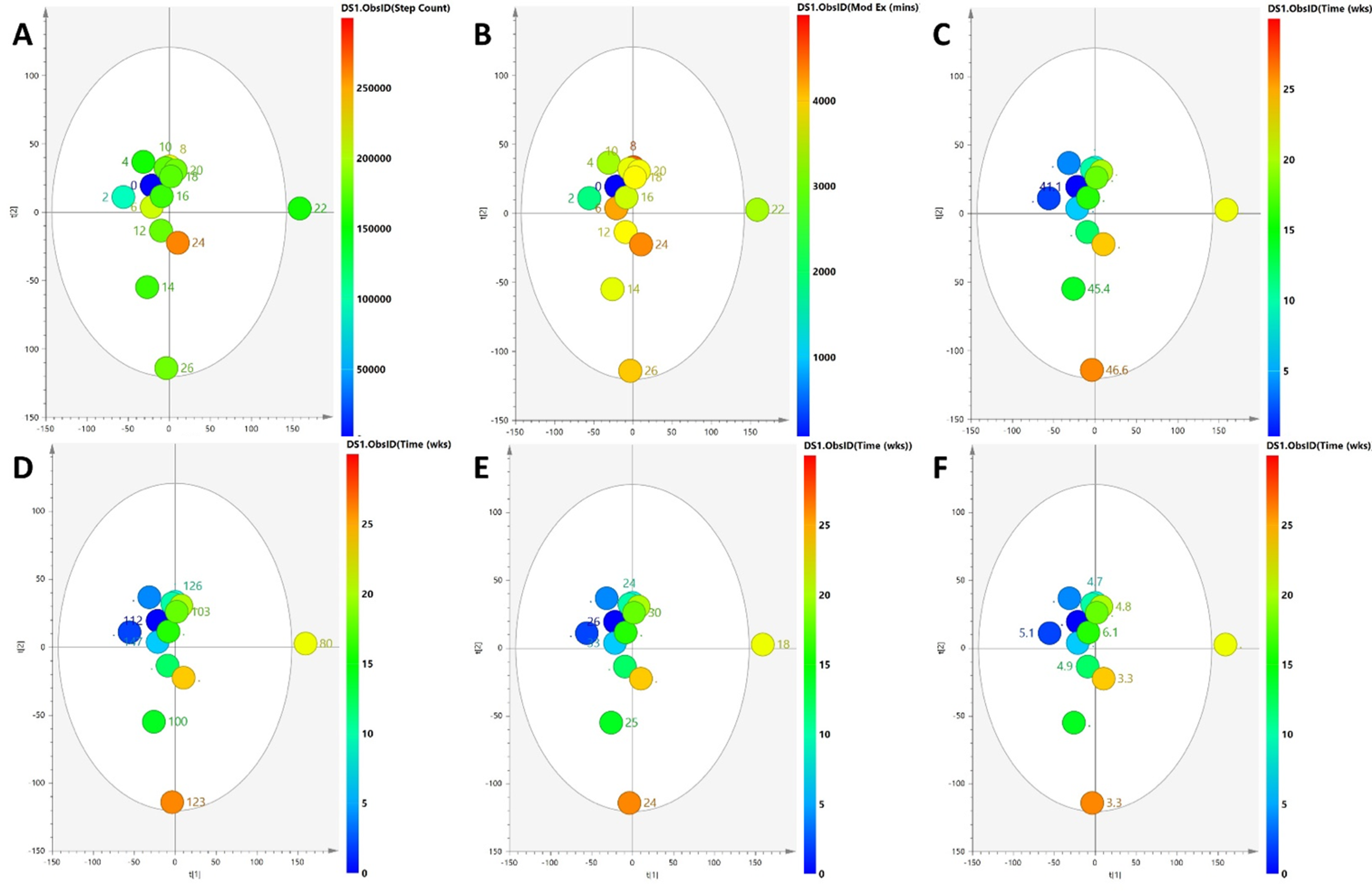

Supplement: Supplementary file 3 — Fig S3 [file TSM2-4-174-s005.tif]

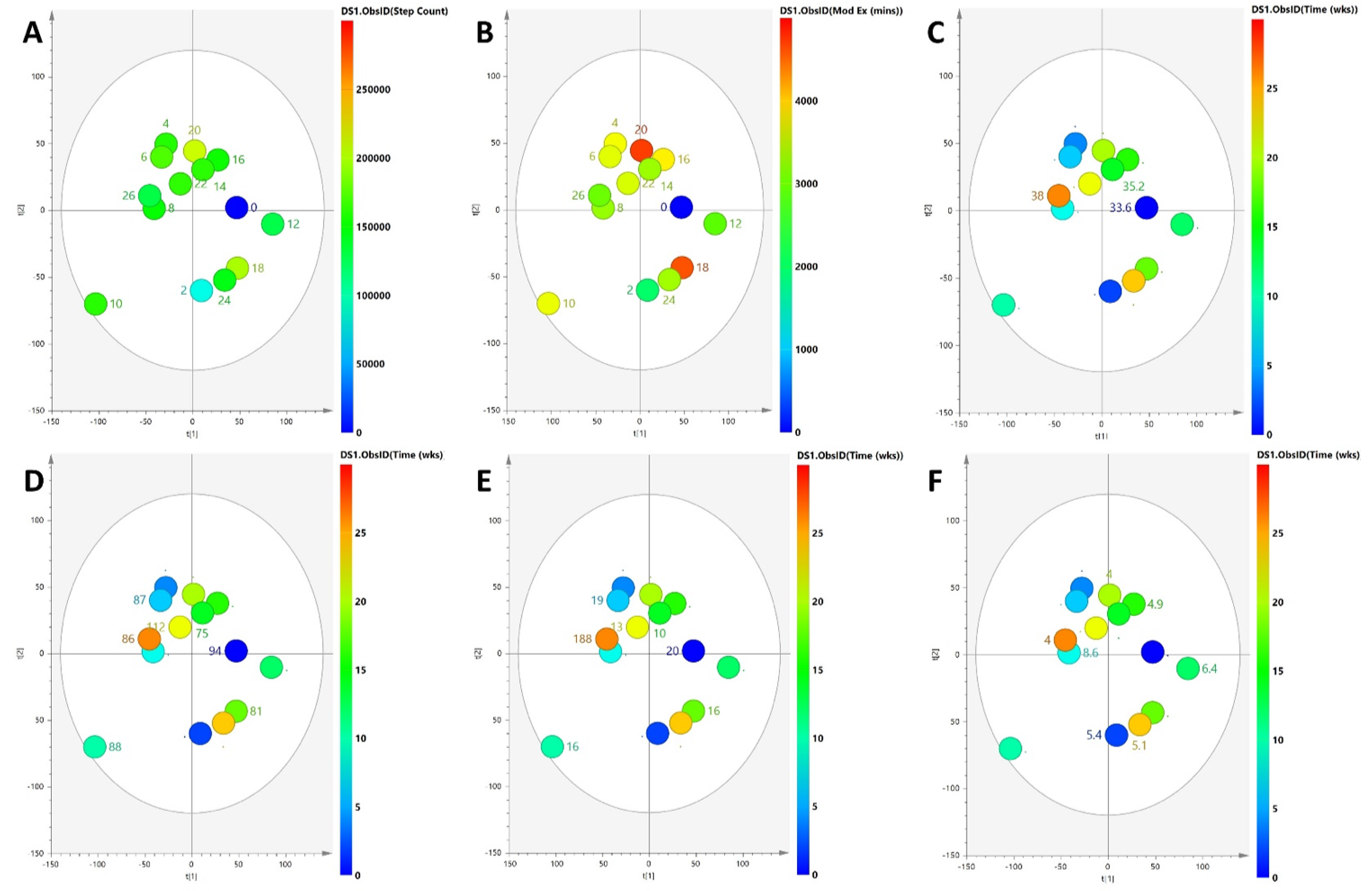

Supplement: Supplementary file 4 — Fig S4 [file TSM2-4-174-s003.tif]
