# Supplementary material for: The effects of sustained fitness improvement on the gut microbiome: A longitudinal, repeated measures case‐study approach
Source: Transl Sports Med. 2020 Dec 13;4(2):174–92. doi: 10.1002/tsm2.215 (PMC8317196; doi:10.1002/tsm2.215)
Supplement: Supplementary file 5 — Table S1 [file TSM2-4-174-s001.docx]

**Table S1** | Inclusion and Exclusion criteria.

| **Inclusion Criteria** |
| --- |
| - 18– 40 year-old (male) |
| - Able to provide informed consent |
| - Good overall medical health |
| - Willing to increase physical activity in a structured manner and to be followed up and measured over a 6-month period |
| - Considered low risk for cardiac events during exercise as guided by the AHA/ACSM’s pre-participation questionnaire |
| **Exclusion Criteria** |
| - Any acute or chronic medical condition including chronic musculoskeletal pain |
| - Personal history of cardiovascular disease of any kind |
| - Family history of cardiovascular disease before the age of 50 |
| - History of total colectomy or significant abdominal surgery |
| - Antibiotic in the last 6 weeks (relative exclusion criteria) |
| - Any medical condition deemed exclusionary by the investigators |
| - A history of substance abuse |
| - Have been on an experimental medication in the last 30 days |
| - On any regular medication |

# Figure legends

**Figure S1** | **Gut microbiome bacterial log Fold Change (logFC).**

Log_2_Fold-change in the marathoner (**A**) and the triathlete (**B**) from the initial time point pre-exercise to post-exercise (log_2_(T14/T0-1)) shows marathoner LogFC lower overall compared to triathlete. Both individuals showed overall species abundance increasing over time. (**C**) Interleuken-6 (IL-6) concentrations every 2 weeks for 6 months.

**Figure S2** | **PCA-Time trajectory scores plots of urinary 1H-NMR profiles colored according to BMI and linear regression to BMI of both volunteers’ urinary UPLC-MS datasets.** Metabolic changes are associated with a reduction in BMI over time. (**A**) Participant 1 exhibited a stable reduction in BMI over the 26 weeks of increased physical activity. (**B**) Participant 2’s reduction in BMI was not constant over time, wherein weeks 10-14 saw the greatest reduction in BMI prior to a gradual increase from weeks 16-26. Ability of the models to account for variation within the datasets is described by R2(X).

**Figure S3** | **PCA-time trajectories of the marathoner’s 1H-NMR urinary dataset colored and labelled according to read-outs of exercise, fitness, and diet**. PCA-time trajectory score plots were colored according to step count and moderate exercise (Mod Ex) in minutes and labelled by time (weeks) (**A**, **B**, respectively). PCA score plots were labelled according to VO2max and colored according to time (**C**). PCA-time trajectories were labelled according to average protein intake (g), average fiber intake (g) and average portions of fruit and vegetables consumed per day (**D**, **E**, **F**, respectively).

**Figure S4** **| PCA-time trajectories of the triathlete’s 1H-NMR urinary dataset colored and labelled according to read-outs of exercise, fitness, and diet**. PCA-time trajectory score plots were colored according to step count and moderate exercise (Mod Ex) in minutes and labelled by time (weeks) (**A**, **B**, respectively). PCA score plots were labelled according to VO2max and colored according to time (**C**). PCA-time trajectories were labelled according to average protein intake (g), average fiber intake (g) and average portions of fruit and vegetables consumed per day (**D**, **E**, **F**, respectively).
